# ID 49 - Análise de Custo-Efetividade da Consulta de Enfermagem Baseada no Modelo Sunrise para Indivíduos com Diabetes Mellitus

**DOI:** 10.5327/2237-9622.2025.v34s1.49

**Published:** 2025-11-25

**Authors:** Cristiano Bertolossi Marta, Thais Braga Meira; Antônio Augusto Freitas Peregrino; Roberto Carlos Lyra da Silva; Renata Braga Meira; Tathiana dos Santos Gomes

**Keywords:** diabetes, Avaliação de Tecnologia em Saúde, tecnologia assistencial, enfermagem

## Abstract

**Introdução:**

A problemática deste estudo gira em torno dos modelos utilizados para a consulta de enfermagem, que não contemplam (como deveriam) o aspecto cultural dos pacientes. Essa lacuna pode comprometer a adesão ao tratamento e, portanto, aumentar os impactos negativos e as consequências impostas pela carga da doença quando o indivíduo não está compensado, sem contar o aumento de custos e a morbimortalidade. Os objetivos: Realizar uma análise de custo-efetividade da utilização ambulatorial do instrumento para a consulta da enfermagem baseada no modelo Sunrise para indivíduos com DM tipos 1 e 2; Estimar a razão de custo-efetividade incremental na redução de internação; e Estimar os custos diretos com o tratamento ambulatorial do diabetes em pacientes com DM tipos 1 e 2 em um instituto referência na temática em tela no estado do Rio de Janeiro.

**Método:**

O método utilizado é o quantitativo. O estudo está de acordo com o tipo Avaliação Econômica Completa em saúde na forma custo-efetividade. Analisamos dois cenários alternativos e mutuamente excludentes, isto é, a utilização (ou não) do instrumento para a consulta de enfermagem baseada no modelo Sunrise. De acordo com os estudos de custo-efetividade, as consequências em saúde devem ser aferidas em uma unidade natural de benefício clínico.

**Resultados:**

Ao considerar a utilização do instrumento baseado no modelo Sunrise para a consulta de enfermagem, a Árvore de Decisão após Roll Back demonstrou que a tecnologia obteve uma efetividade com a redução da internação em até 99,81%, a um custo médio de R$ 3.285 (três mil, duzentos e oitenta e cinco reais) no período analisado. Já a utilização do modelo anterior obteve uma efetividade de até 98,76%, a um custo médio de internação de R$ 3.994 (três mil novecentos e noventa e quatro reais).

**Conclusão:**

O desenvolvimento deste estudo permitiu que comprovássemos que a consulta de enfermagem, baseada no modelo Sunrise, é capaz de reduzir os custos, atender aos objetivos propostos mesmo com uma demanda maior de atendimentos, evitar internações e aumentar a adesão ao protocolo de tratamento ambulatorial; tratando-se de uma doença crônica, isso significa reduzir complicações e melhorar a qualidade de vida dos indivíduos. Dessa forma, se a tecnologia do modelo Sunrise fosse implantada nos ambulatórios de Atenção Primária na cidade do Rio de Janeiro – com cerca de 275 mil pacientes com diabetes nas clínicas da família e centros municipais de saúde –, poderíamos evitar 2.598,75 internações em um ano, o que geraria uma economia de R$ 1.843.969,05.

